# Complementary and Alternative Medicine for the Treatment of Abnormal Endometrial Conditions in Women with PCOS: A Systematic Review and Meta-Analysis of Randomized Controlled Trials

**DOI:** 10.1155/2021/5536849

**Published:** 2021-04-29

**Authors:** Jiayu Hu, Wenhua Shi, Jiayue Xu, Shaoxuan Liu, Siya Hu, Wenjing Fu, Jing Wang, Fengjuan Han

**Affiliations:** ^1^Heilongjiang University of Chinese Medicine, Harbin 150040, China; ^2^Fourth Affiliated Hospital of Harbin Medical University, Harbin 150040, China; ^3^People's Hospital of Langfang City, Langfang 65000, China; ^4^First Affiliated Hospital of Heilongjiang University of Chinese Medicine, Harbin 150040, China

## Abstract

**Background:**

Endometrial lesions in patients with polycystic ovary syndrome (PCOS) exhibit complex pathological features, and these patients are at risk of both short-term and long-term complications. Complementary and alternative medicine (CAM), which is gradually becoming more accepted and is believed to be clinically effective, claims to be promising for treating PCOS, and thus its effect on the abnormal endometrium of PCOS patients should be assessed. The present meta-analysis sought to evaluate the efficacy and safety of CAM in treating endometrial lesions in patients with PCOS.

**Methods:**

Randomized trials on CAM were identified in four Chinese and seven English-language databases from their establishment to January 2020. The present study included patients diagnosed with PCOS and abnormal endometrial conditions who underwent CAM therapy independently or in combination with traditional western medicine. Data were extracted, and the Cochrane “risk of bias” tool was used to assess methodological quality. Effects were expressed as the relative risk (RR) or mean difference (MD/SMD) with 95% confidence interval (CI) as calculated with Rev Man 5.3.

**Results:**

A total of 13 randomized controlled trials were included, involving 1,297 PCOS patients treated for endometrial abnormalities. Methodological quality was generally unclear or had a low risk of bias. The trials tested four different types of CAM therapies (i.e., traditional Chinese medicine treatment, acupuncture treatment, traditional Chinese medicine in combination with western medicine treatment, and acupuncture in combination with western medicine treatment). CAM treatment could significantly reduce the endometrial thickness in PCOS patients compared to western medicine alone (SMD −0.88, 95% CI [−0.12, −0.57]; *I*^2^ = 64%). Compared with clomiphene treatment for the induction of ovulation, CAM treatment showed a clear improvement in endometrial thickness during ovulation (SMD 2.03, 95% CI [1.64, 2.02]; *I*^2^ = 48%). Moreover, CAM was more effective than western medicine alone in reducing the endometrial spiral artery pulsatility index. No significant difference was seen between CAM and traditional treatment when these were used to improve traditional Chinese medicine syndrome scores. Acupuncture alone or traditional Chinese medicines (taken orally) in combination with western medicine significantly increased the pregnancy rate of PCOS patients (RR 1.59, 95% CI [1.30, 1.93]; *I*^2^ = 51%, *P* < 0.00001), and CAM was more effective than western medicine alone for improving hormone levels. No serious adverse events were reported in 11 of the 13 trials.

**Conclusions:**

CAM may effectively ameliorate the endometrial condition of PCOS patients, and it can regulate the level of hormone secretion to increase the ovulation rate and the pregnancy rate.

## 1. Introduction

Polycystic ovary syndrome (PCOS) is a gynecological endocrine disease that is characterized by oligo-ovulation, hyperandrogenemia, and hyperinsulinemia. The prevalence of PCOS has been estimated to be 6–12% in women of childbearing age worldwide [1–3], and the rate in China is about 5.6% [4]. PCOS patients are at increased risk for various complications (e.g., insulin resistance and endometrial abnormalities) along with the typical clinical characteristics of polycystic ovaries, sparse ovulation, and abnormal hormone levels. Changes in the endometrium are among the most common clinical manifestations and complications in PCOS patients. The endometrium of PCOS patients tends to exhibit pathological hyperplasia (e.g., simple hyperplasia, complex hyperplasia, or atypical hyperplasia) [5] due to the long-term exposure to estrogen and the lack of regular progesterone antagonism. As indicated from a previous meta-analysis, PCOS patients are at a higher risk of endometrial cancer, suggesting that long-term pathological endometrial hyperplasia contributes strongly to the development of endometrial cancer [6]. Likewise, PCOS patients suffering from insulin resistance are likely to experience accelerated proliferation of endometrial cells as well as an increased likelihood of long-term complications. In contrast, PCOS patients with infertility suffer from relatively poor endometrial conditions, thereby significantly reducing their pregnancy rate and live birth rate and adversely affecting their health status and their personal family life. When providing treatments for ovulation induction, variations in uterine receptivity have a significant effect on pregnancy outcome [7]. However, there are some indications that modern medical treatments might adversely affect endometrial receptivity in infertile PCOS patients [8]. CAM, which is commonly used to treat PCOS, has been shown to have a positive effect on controlling patients' weight, body mass index, sleep quality, ovulation rate, quality of life, etc. [9–12]. However, no systematic review or research has been conducted on the effects of CAM on the endometrium in PCOS patients, and the endometrium has rarely been discussed as the main outcome index. Therefore, this study undertook a comprehensive literature search on CAM for endometrial intervention in PCOS patients and carried out a systematic review and meta-analysis to supplement the existing evidence in order to determine the contribution of CAM for endometrium abnormalities in PCOS patients and to underpin the clinical treatment of long-term endometrial complications and infertility. Furthermore, only randomized controlled trials (RCTs) were included in the systematic review.

Because PCOS exhibits obvious heterogeneity and because diagnostic standards vary in different regions, this study only included cases that were diagnosed according to the joint criteria of the European Society of Human Reproduction and Embryology (ESHRE) and the American Society of Reproductive Medicine (ASRM) established in Rotterdam in 2003 [13] or according to the Chinese Health Industry Standard WS330-2011: Diagnosis of Polycystic Ovary Syndrome issued by the Chinese Ministry of Health in 2011 [14]. The types of intervention consisted of CAM methods used alone or in combination with traditional western medicine therapy for PCOS.

The concept of CAM has numerous meanings. This study attempted to include as many types of CAM therapies as possible in the literature retrieval in order to avoid any bias in the results due to the omission of therapies. According to the existing research, this study included the following treatment methods within the scope of CAM to treat PCOS: traditional Chinese medicine (TCM), acupuncture, moxibustion, diet suggestions/restrictions, psychological counseling, exercise therapy, and other known CAM methods for treating PCOS [15–19].

## 2. Method

### 2.1. Search Strategy

A systematic literature search was conducted in four Chinese databases (CNKI, WANFANG, VIP, and SINOMED) and seven English databases (PubMed, EMBASE, Web of Science, ProQuest Research Library, Medline, Elsevier/ScienceDirect, and The Cochrane Library) from their time of establishment to January 2020. In addition, gray literature was searched (e.g., meeting minutes). We searched using different combinations of key words, including “polycystic ovary syndrome”, “endometrium”, “complementary and alternative medicine”, “traditional Chinese medicine”, “acupuncture”, “moxibustion”, “exercise therapy”, and “diet intervention”.

#### 2.1.1. Literature Selection and Data Extraction

Two authors (J. Y. Xu and W. J. Fu) independently checked the full text to identify qualified RCTs, and four authors (J. Y. Hu, J. Y. Xu, S. X. Liu, and S. Y. Hu) collaborated with each other to extract data from the included articles according to the predesigned data table. Any conflicts were resolved through discussions with the third author (F. J. Han). The following items were extracted: year of publication, type of study, funding, inclusion/exclusion criteria, diagnostic criteria, research methods, demographic characteristics of the participants, details of the intervention and control, methods of outcome measurement, and adverse events and outcomes.

#### 2.1.2. Quality Assessment

Two authors (J. Y. Hu and J. Y. Xu) used the “risk of bias tool” [20] to assess the methodological quality of the included RCTs. The RCTs were judged as “low risk”, “high risk”, or “uncertain risk” based on risks involving random sequence generation, assignment concealment, blindness of participants and personnel, blindness of the outcome assessment, incomplete data, selective reporting, and other biases (e.g., drug company funding). Any conflicts were resolved through discussions with the third author (F. J. Han).

### 2.2. Data Analysis

This study used the Rev Man 5.3 software for all data analysis. For continuous data, the mean difference (MD/SMD) and 95% confidence interval (CI) were calculated, while for binary data the relative risk (RR) and 95% CI were calculated. If similar study designs, participants, interventions, controls, and outcome indicators were found, then those trials were included in a meta-analysis. Mega data were generated by descriptive counting. Other data not suitable for combination analysis were qualitatively synthesized. In compliance with the recommendations of the *Cochrane Handbook for Systematic Reviews of Interventions* (Higgins 2011), we used the I^2^ test for statistical heterogeneity. If *I*^2^ is greater than 50%, this indicates that there may be substantial heterogeneity [20], so we used the random effects model for data pooling with significant heterogeneity (*I*^2^ ≥ 50%); otherwise, we used the fixed effect model. If data were available, a subgroup analysis was conducted on the subcategories of CAM and a sensitivity analysis was conducted to explore the impact of the type of RCT (parallel or cross randomized) and the quality of the trial (high or low). If more than ten trials were included in the meta-analysis, a funnel chart was generated to explore possible publication bias.

### 2.3. Outcomes

The main analysis included the treatment outcome indicators as measured by one or more of the following items: endometrial thickness (ovulation or luteal metaphase); endometrial type and ovulation rate detected by ultrasound; the levels of sex hormones (mainly follicle stimulating hormone (FSH), luteinizing hormone (LH), and estradiol (*E*_2_)) as measured by chemiluminescence immunoassay; the number of pregnancies (or pregnancy rate) as measured by the level of human chorionic gonadotropin (HCG) and by ultrasound; and the type, number, and probability of adverse reactions. The secondary results included the endometrial spiral artery pulsatility index (PI) and endometrial spiral artery resistance index (RI) of the spiral uterine artery as detected by ultrasound, the cervical mucus score as measured by the Insler cervical scoring method, the early spontaneous abortion rate, the proportion of participants with ≥50% improvement in symptoms and signs according to the assessment of clinicians, and the number or probability of patients with a TCM syndrome differentiation type showing improvement in the TCM syndrome score.

## 3. Results

**Figure 1:**
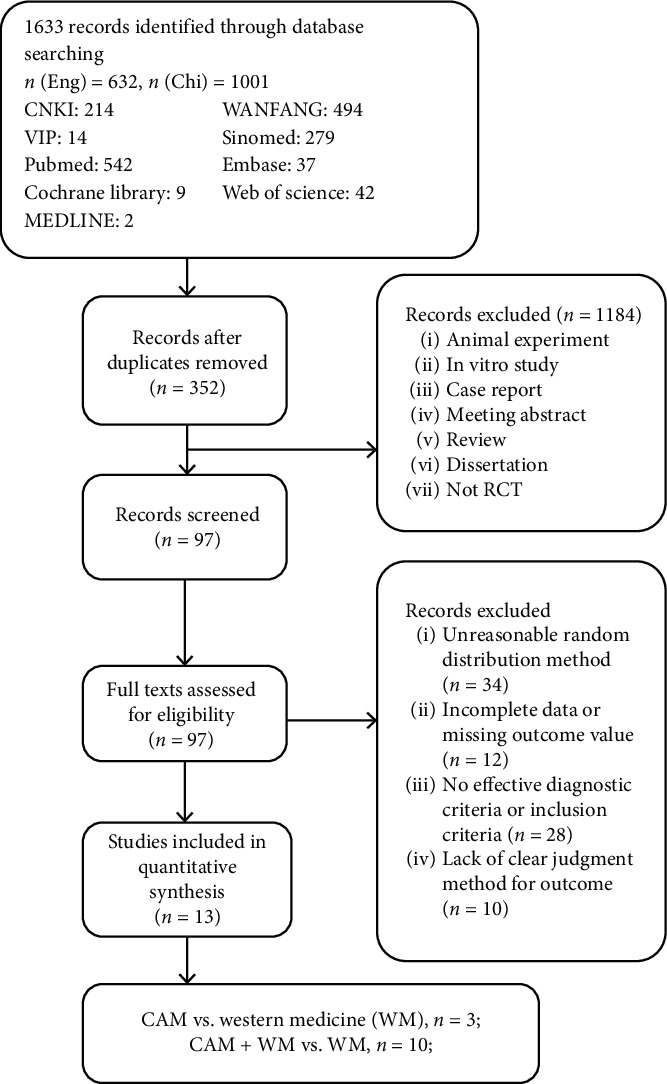
Flow diagram of study selection and different subgroup interventions included in this review.

A total of 1,633 articles were retrieved, including 1,001 in Chinese and 632 in English. After the titles and abstracts were browsed, 1,184 cited trials were excluded due to involving in vitro research, being a dissertation or being non-RCT research, and 352 were duplicates. Among the 97 eligible studies, 34 were excluded for having unreasonable random distribution methods, 12 for having incomplete data or missing outcome indicators, 28 for not mentioning the diagnostic criteria or the inclusion criteria, and 10 for lacking clear methods or criteria for outcome indicators. Finally, 13 trials [21–33] including 1,297 PCOS patients were included in the present review (Figure 1). Twelve of the included trials were in Chinese and one was in English, and all of the studies included patients from mainland China and were carried out by researchers and scholars in mainland China.

The characteristics of the 13 RCTs are shown in Table 1. The sample sizes of the included studies ranged from 56 to 198 participants who ranged in age from 17 to 38 years. In all 13 trials, clinical western medicine treatment for PCOS (e.g., clomiphene and metformin) was used as the western medicine control group, and these included both single drug treatments and multiple drug combinations. In three trials [24, 26, 33], the patients were treated by using CAM alone, with one study using acupuncture alone [33] and two using TCM alone [24, 26]. The remaining 10 trials [21–23, 25, 27–33] used CAM in combination with western medicine. The western medicine control group consisted of clomiphene, metformin, Diane-35, or letrozole alone as well as their combinations with human menopausal gonadotropin (HMG) and HCG.

### 3.1. Bias Risk in the Trials

**Figure 2:**
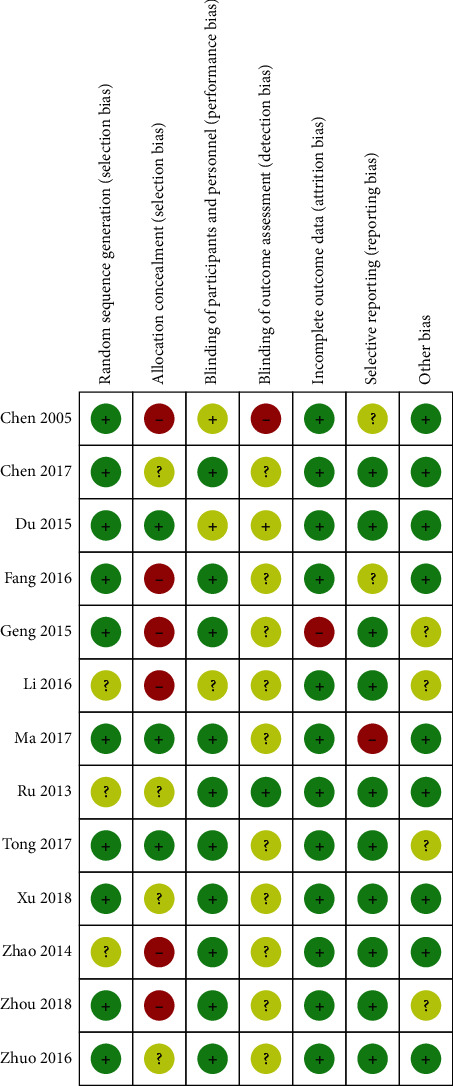
Risk of bias summary.

**Figure 3:**
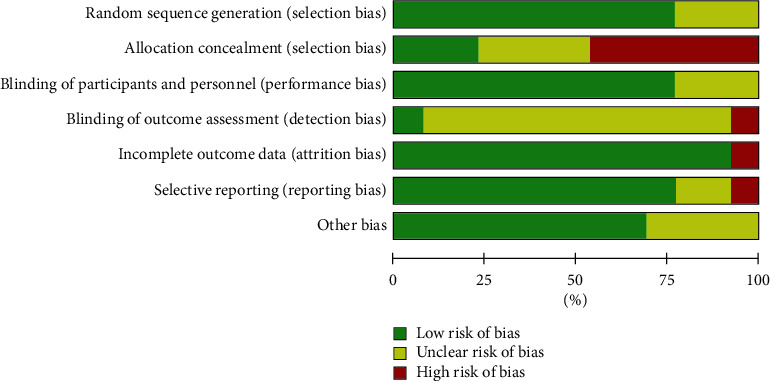
Risk of bias graph.

Three trials [26, 28, 31] were considered to have “unclear” selection bias risk because they only mentioned “random” without describing any specific method of randomization, while the remaining ten trials [21–25, 27, 29, 30, 32, 33] were considered to have a “low” selection bias risk because the methods for generating the random sequence were mentioned (random number table). In one trial [25] considering a “high” reporting bias risk, a case was withdrawn, and this might have led to incomplete follow-up data. The other 12 trials that did not have any case withdrawals included follow-up information [21–24, 26–33] and were considered to have “low” reporting bias risk. Four trials [25, 26, 29, 32] did not report specific details of sample size calculation and were defined as “unclear” risk of other bias (Figures 2 and 3).

#### 3.1.1. Endometrial Thickness

**Figure 4:**
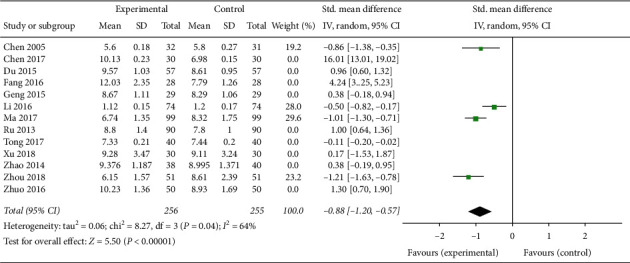
Forest plot of reducing endometrial thickness.

**Figure 5:**
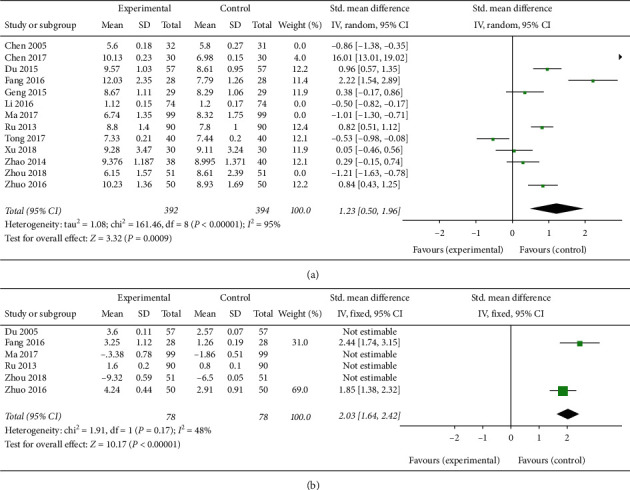
(a) Forest plot of increasing endometrial thickness. (b) Forest plot of increasing endometrial thickness: CAM vs. clomiphene therapy.

Four studies comparing CAM with western medicine treatment and involving 511 patients [21, 26, 27, 32] showed that CAM treatment can significantly reduce the endometrial thickness resulting from abnormal hyperplasia in PCOS patients (SMD −0.88, 95% CI [−0.12, −0.57]; *I*^2^ = 64%) (Figure 4). A comprehensive analysis was performed on the nine articles studying endometrial thickness after CAM alone or in combination with western medicine treatment in PCOS patients with infertility [22–25, 28–31, 33], and the results were found to be highly heterogeneous (SMD 1.23, 95% CI [0.50, 1.96]; *I*^2^ = 95%) (Figure 5(a)). A subgroup analysis was conducted, suggesting that CAM treatment compared with clomiphene therapy for ovulation stimulation significantly increased the endometrial thickness during ovulation (SMD 2.03, 95% CI [1.64, 2.02]; *I*^2^ = 48%) (Figure 5(b)) [24, 33].

#### 3.1.2. Type of Endometrium

**Figure 6:**
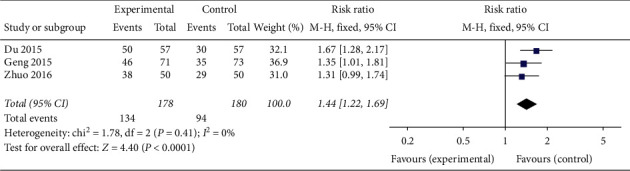
Forest plot of endometrial type A.

**Figure 7:**
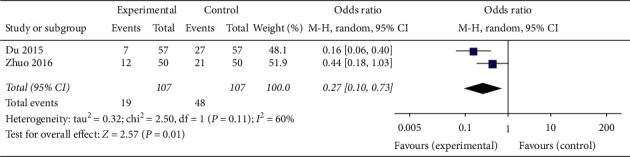
Forest plot of endometrial type *B* + *C*.

In three articles involving 358 patients [23, 25, 33], CAM alone (acupuncture) and CAM (oral TCM) in combination with western medicine effectively increased the number of PCOS patients with type A endometrium compared with clomiphene (RR 1.44, 95% CI [1.22, 1.69]; *I*^2^ = 0%, *P* < 0.0001) (Figure 6). In two articles [23, 33], both acupuncture alone and oral TCM combined with clomiphene significantly downregulated the number of cases of type B and type C endometrium in PCOS patients (RR 0.27, 95% CI [0.10, 0.73]; *I*^2^ = 60%, *P*=0.01) (Figure 7).

#### 3.1.3. PI and RI

**Figure 8:**
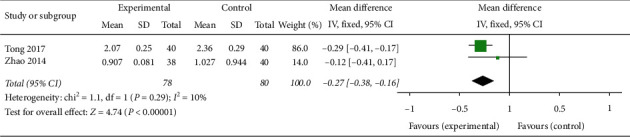
Forest plot of PI.

**Figure 9:**
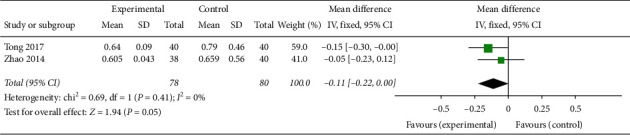
Forest plot of RI.

In two trials involving ovulation stimulation in 158 PCOS patients [29, 31], oral TCM in combination with western medicine could effectively reduce the PI compared with western medicine alone (MD −0.27, 95% CI [−0.38, −0.16]; I^2^ = 10%, *P* < 0.00001) (Figure 8) but had no significant effect on RI (MD −0.11, 95% CI [−0.22, 0.00]; *I*^2^ = 0%, *P*=0.05) (Figure 9).

#### 3.1.4. Hormone Levels

**Figure 10:**
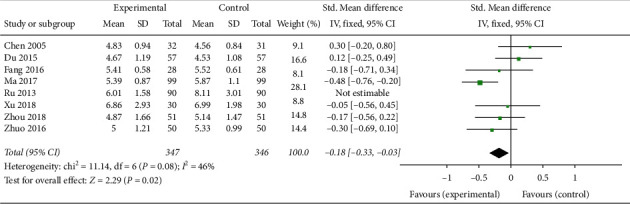
Forest plot of FSH levels.

**Figure 11:**
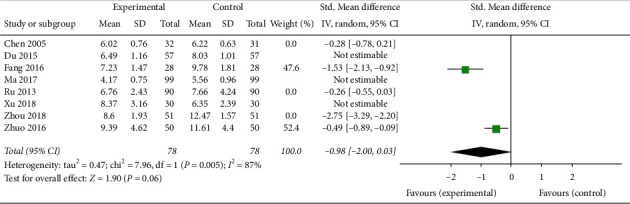
Forest plot of LH levels: TCM or acupuncture + WM vs. WM.

**Figure 12:**
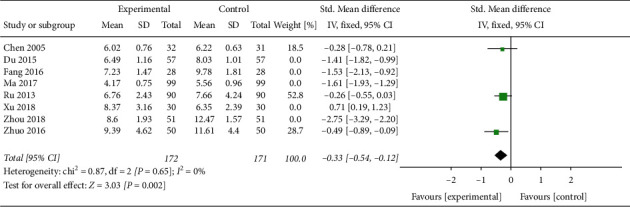
Forest plot of LH levels: acupuncture or TCM + WM vs. WM.

**Figure 13:**
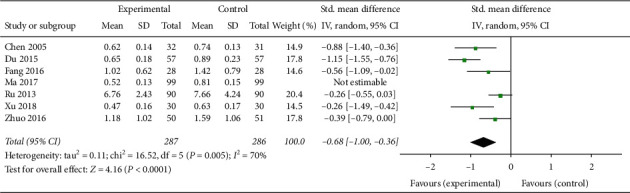
Forest plot of testosterone levels.

**Figure 14:**
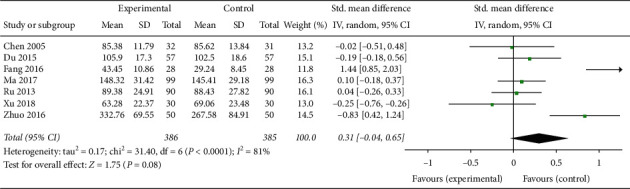
Forest plot of *E*_2_ levels.

Seven trials involving 693 patients [21, 23, 24, 27, 30, 32, 33] showed that CAM alone or CAM in combination with western medicine clearly reduced FSH levels compared with clomiphene and Diane-35 (SMD –0.18, 95% CI [−0.13, −0.33]; *I*^2^ = 46%) (Figure 10). The LH levels in patients treated with TCM or acupuncture in combination with western medicine were not statistically different compared with control patients [24, 33] (Figure 11), while the LH levels in patients treated with acupuncture or TCM in combination with western medicine were significantly improved (SMD −0.33, 95% CI [−0.54, −0.12]; *I*^2^ = 0%, *P*=0.002)(Figure 12) [21, 28, 33]. Both CAM alone (acupuncture or oral TCM) and CAM (acupuncture or oral TCM) in combination with western medicine performed better in decreasing testosterone levels compared with clomiphene and Diane-35 (SMD –0.68, 95% CI [−1.00, −0.36]; *I*^2^ = 70%, *P* < 0.001) (Figure 13) [21, 23, 24, 28, 30, 33]. However, western medicine alone and CAM alone or in combination with western medicine did not lead to significant changes in *E*_2_ levels (SMD 0.31, 95% CI [−0.04, −0.65]; *I*^2^ = 81%, *P*=0.08) [21, 23, 24, 27, 28, 30, 33] (Figure 14).

#### 3.1.5. Number of Dominant Follicles

**Figure 15:**
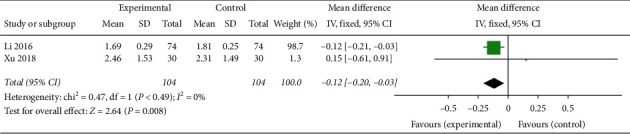
Forest plot of dominant follicle count.

In two trials involving 208 patients, only oral TCM and acupuncture in combination with western medicine effectively increased the number of dominant follicles in PCOS patients after ovulation stimulation treatment (MD −0.12, 95% CI [−0.22, −0.03]; *I*^2^ = 0%, *P*=0.008) [26, 30] (Figure 15).

#### 3.1.6. Number of Ovulation Cases (Ovulation Rate)

**Figure 16:**
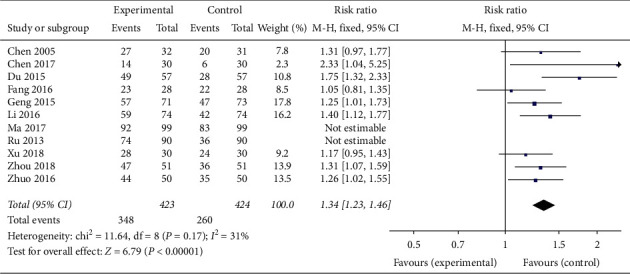
Forest plot of the ovulation rate.

Nine trials involving 847 patients [21–25, 28, 30, 32, 33] showed that the number of ovulation cases (as indicated by signs of ovulation monitored by transvaginal ultrasound over a period of at least 2 months) was significantly increased in those treated with CAM (acupuncture or oral TCM) alone or in combination with western medicine compared to controls (clomiphene or Diane-35) (RR 1.34, 95% CI [1.23, 1.46]; *I*^2^ = 31%, *P* < 0.00001) (Figure 16).

#### 3.1.7. Pregnancy Rate

**Figure 17:**
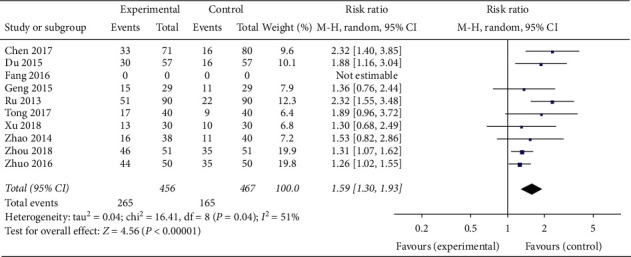
Forest plot of level of the pregnancy rate.

Nine articles involving 923 patients [21, 23, 25, 28–33] showed that acupuncture treatment alone or in combination with western medicine effectively improved the pregnancy rate of PCOS patients (as determined by urine HCG or blood *β*-HCG positivity along with simultaneous ultrasound showing the gestational sac and fetal heart beat) and that oral TCM in combination with western medicine also showed significant improvements in the pregnancy rate of PCOS patients (RR 1.59, 95% CI [1.30, 1.93]; *I*^2^ = 51%, *P* < 0.00001) (Figure 17).

#### 3.1.8. Abortion Rate

**Figure 18:**
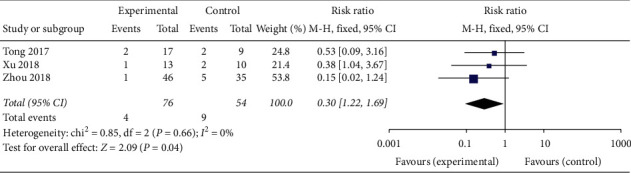
Forest plot of the abortion rate.

Three articles involving 130 patients [29, 30, 32] found that CAM (oral TCM or acupuncture) in combination with western medicine for ovulation simulation was more effective in inhibiting the occurrence of abortion compared with western medicine treatment alone (RR 0.30, 95% CI [0.09, 0.93]; *I*^2^ = 0%, *P*=0.04) (Figure 18).

#### 3.1.9. Ovarian Volume

**Figure 19:**
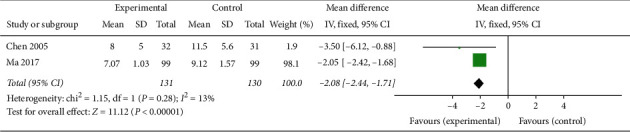
Forest plot of ovarian volume.

As indicated by two articles involving 261 patients [21, 27], the PCOS patients administered oral TCM in combination with western medicine had greater reductions in ovarian volume compared to metformin or Diane-35 ( in MD –2.08, 95% CI [−2.44, −1.71]; *I*^2^ = 13%, *P* < 0.00001) (Figure 19), which was considered a significant improvement in PCOS patients' condition.

#### 3.1.10. Clinical Efficacy

**Figure 20:**
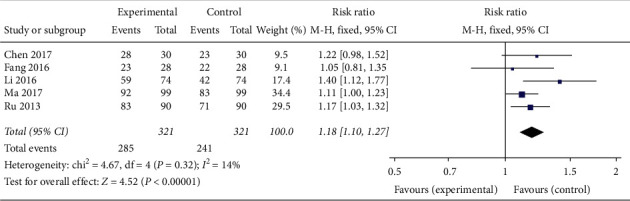
Forest plot of clinical efficacy.

In terms of clinical efficacy, the included trials fell into two groups, namely those that sought to improve the symptoms of PCOS and those that sought to improve the effective pregnancy rate of PCOS patients with infertility. Accordingly, the trials referring to clinical efficacy were integrated, and the clinical efficacy was set as the appearance of effective ovulation in the patients, i.e., the disappearance of mature follicles ≥15 mm or the collapse of the follicle wall as detected through vaginal ultrasound monitoring. Five trials involving 642 patients [22, 24, 26–28] showed that oral TCM alone or in combination with western medicine had an obvious clinical effect in PCOS patients (RR 1.18, 95% CI [1.10, 1.27]; *I*^2^ = 14%, *P* < 0.00001) (Figure 20). Specifically, oral TCM alone [24, 26] was more effective for ovulation simulation compared with western medicine alone (HMG + HCG).

#### 3.1.11. TCM Syndrome Score

**Figure 21:**
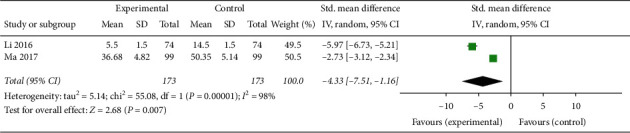
Forest plot of the TCM syndrome score.

Two articles involving 346 patients mentioned the effects of treatments on TCM syndrome differentiation [26, 27], and the TCM syndrome score was determined by a score table as a final indicator of the treatment's effectiveness. However, the results of the two articles for the TCM syndrome score after CAM treatment were not significantly different (SMD −4.33, 95% CI [−7.51, −1.16]; I^2^ = 98%, *P*=0.007) (Figure 21).

#### 3.1.12. Cervical Mucus Score

**Figure 22:**
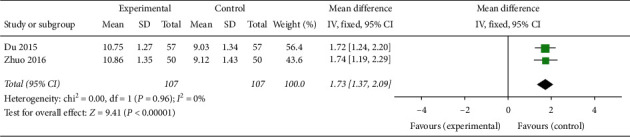
Forest plot of the cervical mucus score.

As suggested by two articles involving 214 patients, [23, 33] CAM (acupuncture) or CAM (oral TCM) combined with clomiphene treatment significantly elevated cervical mucus score compared with clomiphene alone (MD 1.73, 95% CI [1.37, 2.09]; *I*^2^ = 0%, *P* < 0.00001) (Figure 22).

#### 3.1.13. Adverse Reactions

**Figure 23:**
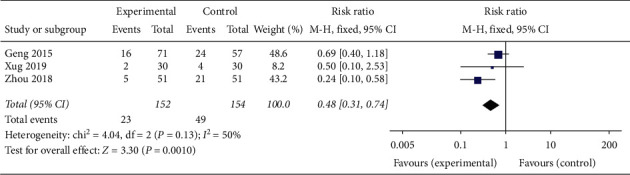
Forest plot of the number of adverse reactions.

Three articles [25, 30, 32] mentioned adverse reactions (e.g., luteinized unruptured follicle syndrome and ovarian hyperstimulation syndrome) during ovulation simulation treatment. Compared with the patients treated with Diane-35 or clomiphene alone, the proportion of adverse reactions in patients administrated with CAM (oral TCM or acupuncture) in combination with western medicine was significantly reduced (RR 0.48, 95% CI [0.31, 0.74]; *I*^2^ = 50%, *P*=0.001) (Figure 23).

One article [21] reported that TCM in combination with Diane-35 and Diane-35 alone effectively reduced the endometrial thickness and ovarian volume and significantly improved the number of dominant follicles. However, after six cycles after discontinuation of treatment, the endometrial thickness and ovarian volume of the western medicine control group were close to the pretreatment status, while the CAM intervention group effectively maintained the normal levels.

In one article, due to the additional intervention of aspirin in both groups, we could not absolutely attribute the effectiveness to CAM in determining the outcome indicators when compared with the western medicine control group [31].

#### 3.1.14. Additional Analysis

Because fewer than 10 trials were contained in each comparison, this study could not conduct meaningful funnel chart analysis to determine publication bias. In addition, the heterogeneity of the endometrial thickness and the LH level reached over 70% (*I*^2^ ≥ 70%), so a subgroup meta-analysis was conducted. Different types of CAM were used in the included RCTs, and this resulted in a certain level of clinical heterogeneity in the results of this study.

## 4. Discussion

This review identified 13 RCTs involving 1,297 PCOS patients with abnormal endometrial status. The normal endometrial stages include proliferation, secretion, and menstrual periods, and thus the thickness of the endometrium changes over the course of the menstrual cycle. Most researchers consider that if the endometrium is extremely thin (<8 mm), this might cause unfavorable condition for embryo implantation and thus result in a low clinical pregnancy rate, and extreme thickness (>16 mm) might also reduce the clinical pregnancy rate [34]. Accordingly, as indicated from the results presented here, CAM therapy (including CAM therapies used alone as well as CAM therapies used in combination with traditional western medicine) is capable of effectively reducing the thickness of the endometrium in pathological hyperplasia, while in PCOS patients with infertility it can increase the thickness of the endometrium during ovulation thereby improving the status of the endometrium, increasing the pregnancy rate, and decreasing the abortion rate. Thus, depending on the patient's condition, CAM can reduce or increase the thickness of the endometrium as needed to promote pregnancy more effectively than traditional western medicine and can help to avoid long-term complications such as endometrial cancer. Compared with western medicine (e.g., clomiphene, Diane-35, and other ovulation simulation treatments), CAM is capable of significantly reducing the adverse reactions associated with ovulation simulation, improving cervical mucus score, increasing ovulation rate, increasing the number of dominant follicles, and increasing the pregnancy rate, thus showing an overall clinical effect of CAM treatments.

Furthermore, this review also focused on the impact of CAM treatment on the hormone levels in PCOS patients. Although *E*_2_ is directly involved in hyperplasia of the endometrium, CAM does not noticeably affect the *E*_2_ level of PCOS patients. This may be related to the small number of articles included. FSH and LH, as gonadotropins, are not directly involved in the cyclic changes of endometrial hyperplasia and secretion, but regulation of their receptors can affect the intracellular function of the glandular epithelium of the endometrium where the receptors are expressed [35]. Gonadotropin receptor levels are positively correlated with the development of endometrial cancer [36], and it is known that exposure to high-level FSH conditions can increase the proliferation, invasion, and metastasis of endometrial cancer cells [37]. The results of the present analysis strongly suggest that CAM is capable of effectively reducing the serum FSH level in PCOS patients and thus reducing the risk of endometrial cancer. Moreover, this study found that CAM can reduce the serum testosterone level in PCOS patients with endometrial abnormalities. The elevation of testosterone is considered one of the common symptoms in PCOS patients, and testosterone, as a type of androgen, suppresses the autoimmune system [38] and can lead to cancer. Patients with endometrial cancer and endometrial adenoma often show elevated levels of testosterone [39, 40], and thus by reducing the level of testosterone CAM might prevent long-term complications such as endometrial cancer in PCOS patients.

The normalization of the endometrium, which is a necessary condition for successful pregnancy, is a critical outcome indicator for PCOS patients. Type A endometrium is more conducive to pregnancy due to its richer blood supply and higher receptivity than types B and C [41], and this review found that CAM significantly increases the occurrence of type A endometrium thus suggesting that CAM can improve the pregnancy rate in PCOS patients. By ameliorating abnormalities of the endometrium, CAM positively affected the pregnancy rate and live birth rate in PCOS patients, thus further proving that PCOS patients with complications (e.g., infertility) can be effectively treated with CAM.

According to the existing literature, the abnormal proliferation of the endometrium in PCOS patients shows a close positive correlation with the expression of prolactin and its receptors [42]. One of the included RCTs involved 80 PCOS patients and 80 matching controls and showed that the visfatin protein in the endometrial tissue of PCOS patients was highly upregulated and that the phosphorylation of AKT and ERK1/2 was also significantly increased, thus indicating that the malignant transformation of the endometrium in PCOS patients might be associated with the visfatin protein and the activation of the AKT and ERK1/2 signaling pathways [43]. Moreover, the abnormal state of the endometrium in PCOS patients can be manifested as insulin resistance contributing to abnormal glucose metabolism [44]. It has been reported that the endometrium of PCOS patients might suffer from abnormal amino acid metabolism in the tryptophan, tyrosine, and phenylalanine pathways, thereby causing abnormal cell proliferation and decreasing endometrial receptivity [45]. In the trials included here, however, none studied the effects of CAM on the factors that have been reported to cause endometrial abnormalities, and thus it remains unclear if CAM can effectively ameliorate the abnormalities of the endometrium after the related pathogenic factors mentioned above have been appropriately regulated.

While the endometrial problems in PCOS patients have aroused huge attention [46–49], CAM has not been valued as a primary method to improve the endometrial status in these patients. However, CAM has been found to be increasingly employed in controlling body weight and improving hormone levels and ovulation rates in PCOS patients [10, 50, 51]. Most of the studies included here have bias risk in numerous areas (e.g., distribution concealment, blindness, data loss, and sample size calculation), and thus the effectiveness of CAM remains unclear.

### 4.1. Strengths and Limitations

The present meta-analysis systematically evaluated the efficacy and safety of CAM in treating endometrial lesions in patients with PCOS. Although this study searched as many trials as possible, we still cannot be sure that we have covered all the evidence, and there might still be unanalyzed or unpublished data that might influence our conclusions. Additionally, in the retrieval process factors such as exercise, diet intervention, and psychological influences were not considered, so omissions and deficiencies might have occurred in the retrieval of RCTs studying CAM therapy. The final 13 RCTs included here focused on TCM treatment, and the research subjects and researchers almost exclusively originated from mainland China, and thus there was a lack of research information about other regions, which may have caused other bias. Moreover, most of the included RCTs lacked clear double-blind design methods, resulting in the low quality of the included RCTs. Furthermore, due to the statistical heterogeneity and variability of the CAM methods, subgroup meta-analysis, meaningful sensitivity analysis, and funnel chart analysis could not be conducted. Thus the present systematic review is limited in terms of the validity and universality of its conclusions, and this suggests that future RCTs should be designed as multicenter, double-blind placebo-controlled trials with more indicators of effectiveness, and they should be reported in accordance with the CONSORT (Consolidated Standards for Reporting Trials) criteria [52].

## 5. Conclusion

This systematic review suggests that CAM has potential for improving endometrial thickness, endometrial type, serum hormone level, and pregnancy rate in PCOS patients. However, due to the limited quantity and the general low quality of the methodology of the included trials, more in-depth research is required before CAM can be applied more widely in clinical practice. Thus more rigorous double-blind, placebo-controlled trials should be conducted to confirm the efficacy of CAM in improving endometrial condition in PCOS patients.

## Figures and Tables

**Table 1:** Characteristics of included randomized clinical trials on CAM therapies for PCOS abnormal endometrial conditions.

Study ID	Sample size	Age	Comparisons	Outcome	Follow-up
*CAM vs. WM, 3 studies*					
Fang et al. [24]	T: 28°C: 28	T: 27.54 ± 3.65 years C: 28.24 ± 4.36 years	Chinese medicine prescription vs. clomiphene (3 m)	①②③⑧	NR
Li [26]	T: 74°C: 74	T: 27.8 ± 3.50 years C: 28.0 ± 3.50 years	Chinese medicine prescription + progesterone capsule (under certain conditions) vs. HMG + HCG + progesterone capsule (under certain conditions) (3–6 m)	①③⑥⑨	NR
Zhuo [33]	T: 50°C: 50	T: 29 ± 5 years C: 28 ± 5 years	Acupuncture vs. clomiphene (3 m)	①②④⑤⑪⑫	NR

*CAM* *+* *WM vs. WM, 10 studies*					
Chen et al. [22]	T: 30°C: 30	T: 28.63 ± 0.73 years C: 30.13 ± 0.75 years	Ding Kundan + clomiphene + HMG + dydrogesterone (under certain conditions) vs. clomiphene + HMG + dydrogesterone (under certain conditions) (1 m)	①③④⑤⑩	1 m
Chen [21]	T: 32°C: 31	T: 28.63 ± 0.73 years C: 30.13 ± 0.75 years	Yougui Pill (adjusted according to conditions) + Diane-35 vs. Diane-35 (3 m)	①②⑤⑦	6 m
Du [23]	T: 57°C: 57	T: 29.4 ± 5.3 years C: 28.6 ± 5.7 years	Chinese medicine prescription + ethinyl estradiol + clomiphene citrate tablets + HMG vs. ethinyl estradiol + clomiphene citrate tablets + HMG (3 m)	①②④⑤ ⑪ ⑫ ⑬⑯	NR
Hongling and Limian [25]	T: 29°C: 29	T: 25.8 ± 1.8 years C: 26.2 ± 2.4 years	Traditional Chinese medicine + clomiphene citrate tablets + estradiol valerate + HCG vs. clomiphene citrate tablets + estradiol valerate + HCG (3 m)	①④⑤⑩⑪	3 m
Ma et al. [27]	T: 99°C: 99	T: 28.7 ± 5.1 years C: 27.4 ± 14.8 years	Chinese medicine prescription + metformin vs. metformin (3 m)	①②③⑥⑦	NR
Ru et al. [28]	T: 90°C: 90	T: 26.8 ± 4.4 years C: 26.5 ± 5.0 years	Chinese medicine prescription + clomiphene vs. clomiphene (3 m)	①②③④⑤	NR
Tong et al. [29]	T: 40°C: 40	T: 30.58 ± 3.82 years C: 30.23 ± 3.53 years	Chinese medicine prescription + clomiphene + HCG (under certain conditions) + dydrogesterone (under certain conditions) vs. Clomiphene + HCG (under certain conditions) + dydrogesterone (under certain conditions) (3 m)	①④⑤⑭⑮	NR
Xu and Zho [30]	T: 30°C: 30	T: 25.7 ± 4.0 years C: 25.8 ± 4.2 years	Acupuncture + Diane-35 + HMG + HCG vs. Diane-35 + HMG + HCG (2m)	①②④⑤⑨⑩ ⑭	NR
Zhao et al. [31]	T: 38°C: 40	T: 26.21 ± 3.37 years C: 26.30 ± 3.38 years	Letrozole tablets + Tiao Jing Cu Yun pills + aspirin vs. letrozole tablets (only 1 m concluded)	①④⑮	NR
Wenqin and Dianzhou [32]	T: 51°C: 51	T: 28.6 ± 5.14 years C: 29.4 ± 6.14 years	Tiao jing cu yun pill + clomiphene citrate capsules + HCG vs. clomiphene + HCG (3 m)	①②④⑤⑩⑭	NR

① Endometrial thickness, ② hormone levels, ③ clinical efficacy, ④ pregnancy rate (number of cases), ⑤ ovulation rate (number of cases), ⑥ TCM Syndrome Score, ⑦ ovarian volume, ⑧ dominant follicle size, ⑨ dominant follicle count, ⑩ adverse reactions, ⑪ endometrial type, ⑫ cervical mucus score, ⑬ number of menstrual recovery cases, ⑭ abortion rate, ⑮ endometrial blood flow coefficient (PI, RI, etc.), and ⑯ LP leptin.*∗*HMG: human menopausal gonadotropin, HCG: human chorionic gonadotropin, m: month, and NR: no report.

## Data Availability

The data for this study are available from the corresponding author upon request.
